# Evaluation of Morbidity and Mortality in Iatrogenic Colonic Perforation During Colonoscopy: A Comprehensive Systematic Review and Meta-Analysis

**DOI:** 10.7759/cureus.73302

**Published:** 2024-11-08

**Authors:** Ajibola A Adebisi, Daniel E Onobun, Adeola Adediran, Reginald N Ononye, Ethel O Ojo, Adedayo Oluyi, Ayotunde Ojo, Stephen Oputa

**Affiliations:** 1 General Surgery, Epsom and St Helier University NHS Foundation Trust, London, GBR; 2 Orthopaedics and Trauma, Warwick Hospital, South Warwickshire University NHS Foundation Trust, Warwick, GBR; 3 Public Health, University of Ibadan, Ibadan, NGA; 4 Urological Surgery, Glan Clwyd Hospital, Bodelwyddan, GBR; 5 Trauma and Orthopaedics, University Hospital Leicester, Leicester, GBR; 6 General Surgery, Tunbridge Wells Hospital, Kent, GBR; 7 General Surgery, University of Nigeria, Enugu, NGA

**Keywords:** clinical factors, comorbidities, iatrogenic colonic perforation (icp), morbidity, mortality, operator experience, patient-specific factors, perforation severity, routine colonoscopy, surgical intervention

## Abstract

This systematic review and meta-analysis explores the clinical and patient-specific factors contributing to increased morbidity and mortality following iatrogenic colonic perforation (ICP) during routine colonoscopy. A comprehensive search of Scopus, PubMed (Medline), Embase, and Google Scholar was conducted, reviewing studies published between 2010 and 2024. Data were synthesized through thematic analysis for qualitative data and meta-analysis for quantitative outcomes.

This study found the overall perforation rate during colonoscopy to be between 0.019% and 0.66%. The average age of patients was 68.91 years. The mortality rate was approximately 11%. Older patients had a higher risk of perforation and an increased mortality rate. Therapeutic colonoscopy carries a significantly higher risk of perforation compared to diagnostic colonoscopy. Diagnostic colonoscopies were more likely to cause larger perforations, which, in turn, carry greater morbidity than smaller perforations. The most common sites of perforation are the sigmoid colon and the rectosigmoid junction. The leading causes of perforation include diverticulitis, malignancy, abnormal sigmoid anatomy, and inflammatory bowel disease. About half of the perforations were discovered during or immediately after the procedure. The most common surgical interventions were resection with primary anastomosis and Hartmann’s procedure. Operators with limited experience were more likely to cause perforations and experience delayed recognition of the issue.

The results suggest that both clinical and patient-specific factors play critical roles in determining outcomes for patients experiencing ICP during routine colonoscopy. Early detection, timely intervention, and operator experience are key to improving patient survival and reducing complications. Furthermore, the study highlights the need for ongoing research to refine patient selection criteria and enhance postoperative care protocols, particularly for high-risk populations such as the elderly and those with significant comorbidities.

This review underscores the importance of heightened vigilance in colonoscopy procedures, especially among vulnerable populations. Future research should focus on improving diagnostic protocols and treatment strategies to minimize the risks associated with ICP and enhance patient safety in routine colonoscopy practices.

## Introduction and background

Iatrogenic colonic perforation (ICP) remains a life-threatening complication and one of the most serious adverse events associated with colonoscopy. Although relatively rare, the consequences of ICP can be severe, leading to complications such as intra-abdominal sepsis, stoma formation, prolonged hospitalization, and even death [1, 2]. Despite advances in colonoscopy techniques, ICP continues to carry high mortality and morbidity rates, estimated to be about 25% and 40%, respectively, primarily due to the leakage of bowel contents into the peritoneal cavity, which can result in intra-abdominal sepsis, septic shock, abscess formation, and multi-organ failure [3-6]. The frequency of ICP during colonoscopy has been reported to range from 0.1% to 0.8% in diagnostic procedures and 0.15% to 3% in therapeutic colonoscopies, with the risk being particularly elevated in elderly patients, where it can rise to 1.9% [7, 8].

The underlying causes of ICP often include diverticulitis, malignancies, ischemia, infections such as wound infections or postoperative fever, inflammatory bowel disease, and iatrogenic factors related to the procedure itself [2, 9-11]. These varied etiologies make ICP a complex condition to manage and predict. This systematic review and meta-analysis aim to explore the clinical and patient-specific factors that contribute to increased morbidity and mortality following ICP during routine colonoscopy. By identifying and analyzing these factors, this study seeks to provide a comprehensive understanding of the risks associated with ICP and offer insights into how these risks can be mitigated in clinical practice.

Although ICP is uncommon, its potential to cause life-threatening conditions presents a significant challenge in clinical settings. Despite improvements in surgical techniques and increased awareness of the risks involved in colonoscopy, ICP continues to pose substantial challenges for healthcare providers [6, 12-15]. This study focuses on the clinical and patient-specific factors that influence morbidity and mortality in ICP cases, with the aim of identifying strategies to reduce these adverse outcomes. Understanding these factors is crucial for refining clinical guidelines, improving patient safety, and reducing the incidence and severity of ICP-related complications.

The key research question guiding this review is: What are the clinical and patient-specific factors associated with increased morbidity and mortality following ICP in routine colonoscopy? The objectives of this study are to analyze the clinical factors contributing to higher morbidity and mortality rates, evaluate the impact of patient-specific factors on these outcomes, and assess the differences in morbidity and mortality rates across various patient profiles. By achieving these objectives, the review aims to enhance clinical practice and improve patient outcomes in cases of ICP.

## Review

Methodology

Study Design

This systematic review and meta-analysis aimed to identify the clinical and patient-specific factors associated with increased morbidity and mortality in patients experiencing ICP during routine colonoscopy. The study followed the guidelines established by the Preferred Reporting Items for Systematic Reviews and Meta-Analyses (PRISMA) 2020 statement and the Quality of Reporting of Meta-analyses (QUORUM) to ensure transparency, accuracy, and reproducibility of the review process.

Search Strategy

A comprehensive literature search was performed to identify relevant studies published between 2010 and 2024. Four major electronic databases were searched: PubMed (Medline), Scopus, Embase, and Google Scholar. The search strategy was designed to capture all studies reporting on clinical and patient factors associated with ICP during colonoscopy. Boolean operators "AND" and "OR" were applied to combine search terms and retrieve relevant literature. The search terms included "iatrogenic colonic perforation", "routine colonoscopy", "clinical factors", "patient-specific factors", "increased morbidity", and "increased mortality", used in varying combinations, such as "morbidity of iatrogenic colonic perforation", "mortality of iatrogenic colonic perforation", and "prognostic factors of iatrogenic colonic perforation".

To ensure inclusiveness and minimize selection bias, the search strategy was adapted for each database using both Medical Subject Headings (MeSH) terms and free-text keywords. Additional hand-searching of the references of included studies was conducted to identify any potentially relevant papers not captured by the initial search. The detailed search strategy is provided in Table 1.

**Table 1:** Search Strategy

Key Terms	Possible Alternative Search Keywords
Clinical factors	Clinical characteristics, clinical parameters
Patient-specific factors	Patient factors, Patient characteristics, prognostic factors, patient-specific parameters
Increased morbidity	High rate of morbidity
Increased mortality	High rate of mortality
Iatrogenic colonic perforation	Colonic perforation, colorectal perforation, colon perforation
Routine colonoscopy	Colonoscopy, therapeutic colonoscopy, diagnostic colonoscopy

Inclusion and Exclusion Criteria

The eligibility criteria were established before the review process to ensure consistency and transparency. Studies were included if they met the following criteria: patients experiencing ICP during routine colonoscopy were the population studied; the intervention focused on clinical and patient-specific factors related to ICP; randomized controlled trials (RCTs), cohort studies, case-control studies, and observational studies that used either qualitative, quantitative, or mixed methods were considered. The outcome of interest included studies reporting morbidity and mortality rates associated with ICP. Only studies published in English between 2010 and 2024 were included to cover a 15-year period.

Studies were excluded if they did not focus on the clinical and patient-specific factors related to ICP. Reviews, meta-analyses, conference proceedings, editorials, book chapters, and non-peer-reviewed articles were also excluded, as were studies published in languages other than English or before 2010. The inclusion and exclusion criteria are summarized in Table 2.

**Table 2:** Inclusion and Exclusion Criteria

Criteria for Inclusion	Criteria for Exclusion
Studies reporting on the clinical and patient factors associated with increased morbidity and mortality in patients experiencing iatrogenic colonic perforation	Studies that do not focus on the clinical and patient factors associated with increased morbidity and mortality in patients experiencing iatrogenic colonic perforation
Studies carried out from 2010 to 2024	Studies carried out before 2010
Studies with clear and reproducible methods and clear outcomes. Journals/articles that used qualitative, quantitative method, or mixed methods	Systematic reviews, meta-analyses, chapters, editorials, books, conference proceedings, and reviewed journals
Publication type and language: peer-reviewed journals and English	Publication in other languages, other studies that are not peer-reviewed

Study Selection Process

The study selection process adhered to PRISMA guidelines. An in-depth electronic search was conducted across the e-databases PubMed, Scopus, Embase, and Medline to identify relevant papers published between 2010 and 2024. Search strings were tailored for each database, and Google Scholar was used to locate additional relevant literature. Initially, the titles and abstracts of all identified studies were screened to determine their relevance based on the inclusion and exclusion criteria. Full-text articles of potentially eligible studies were then retrieved for detailed assessment. Duplicate studies were removed using the Mendeley reference manager, which also facilitated the organization and management of the retrieved references.

**Figure 1:**
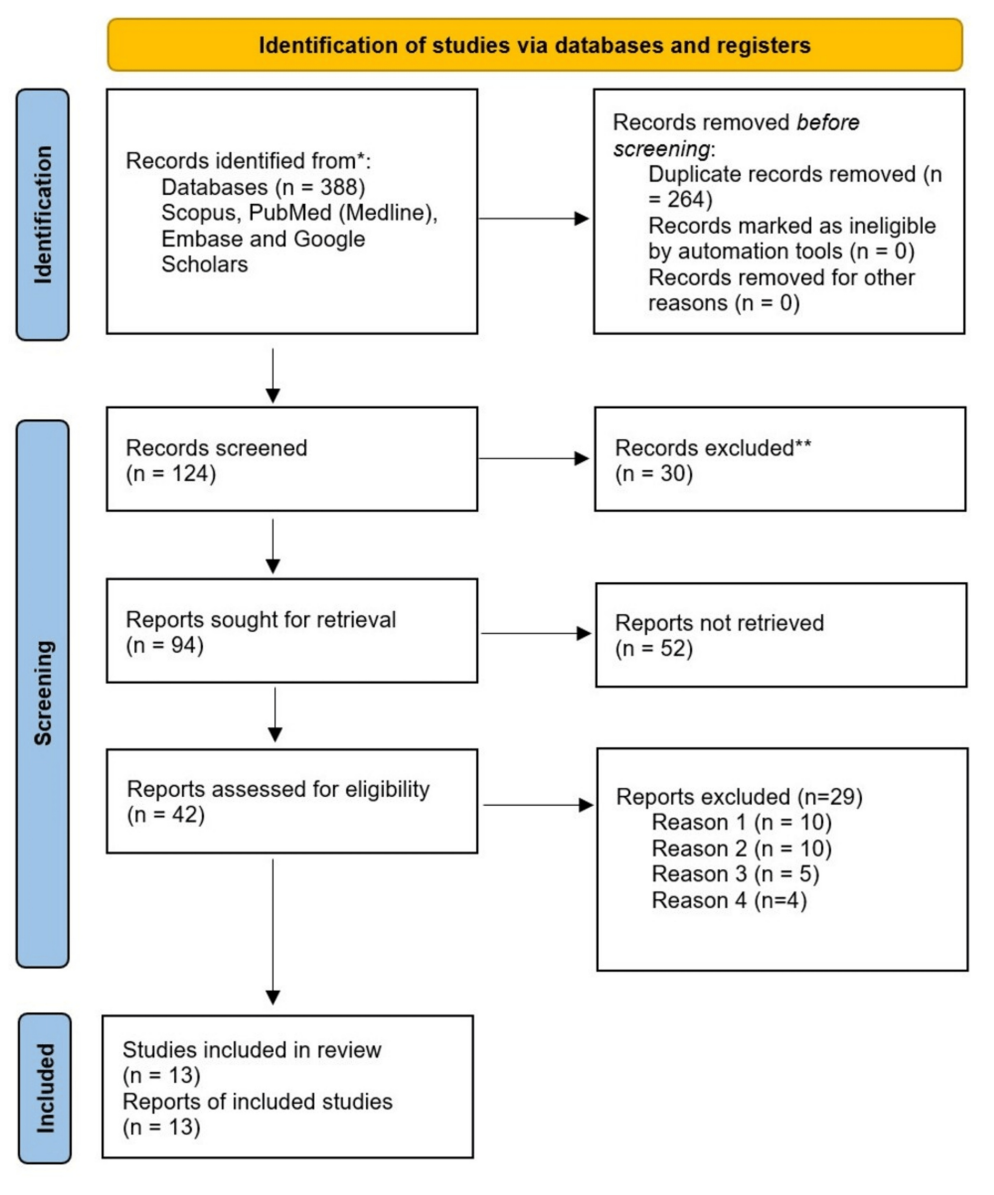
PRISMA Flow Chart PRISMA: Preferred Reporting Items for Systematic Reviews and Meta-Analyses.

The study selection process involved three independent reviewers who evaluated each study's eligibility. Discrepancies were resolved through discussion, and if disagreements persisted, a fourth reviewer was consulted. The PRISMA flowchart (Figure 1) illustrates the selection process, including the number of studies identified, screened, assessed, and ultimately included in the final analysis.

Data Analysis

This review employed both thematic analysis for qualitative data and meta-analysis for quantitative data. Thematic analysis was performed to identify patterns and themes related to clinical and patient-specific factors from the included qualitative studies. The extracted qualitative data were reviewed to identify and define themes, which were subsequently named and presented in the results. This approach facilitated the identification of common clinical and patient-specific factors across the studies.

For the quantitative data, a meta-analysis was conducted by calculating the weighted pooled estimate for interventions reported in at least two distinct studies. The pooled estimates were displayed in forest plots, which illustrated confidence intervals and odds ratios for each study. Due to expected heterogeneity among the studies, a random-effects model was applied. The primary outcome measures were the pooled morbidity and mortality rates, while secondary outcome measures included the length of hospital stay, complication rates, and severity of perforation.

Heterogeneity among the included studies was assessed using the I² statistic, with values above 50% indicating significant heterogeneity. Publication bias was evaluated using funnel plots and Egger's test. When publication bias was detected, the trim-and-fill method was applied to adjust for potential biases.

The pooled estimates for morbidity and mortality rates were calculated using odds ratios (ORs) and 95% confidence intervals (CIs). Forest plots were generated to visually represent the meta-analysis results, with individual study effect sizes depicted as squares and the overall effect size represented by a diamond. Sensitivity analyses were performed by excluding low-quality studies to evaluate the robustness of the findings.

Results

Study Selection

A total of 388 studies were identified through the initial search of databases, including PubMed, Scopus, Embase, and Google Scholar, as well as reference screening. After removing 264 duplicates and studies that did not meet the inclusion criteria, 124 studies were screened based on their titles and abstracts. Of these, 82 studies were excluded for not meeting the eligibility criteria. Full-text reviews were conducted on 42 studies, with 29 being excluded due to reasons such as inadequate focus on clinical and patient-specific factors, insufficient data on morbidity or mortality, or lack of relevance to ICP. Ultimately, 13 studies were included in the systematic review and meta-analysis.

The PRISMA flow diagram in Figure 1 illustrates the study selection process, detailing the number of studies excluded at each stage.

Study Characteristics

The 13 studies included were published between 2010 and 2024 and conducted across various countries, including Korea, Turkey, Portugal, Lithuania, Japan, China, and the United States. The studies included a range of designs, from retrospective cohort studies to prospective observational designs. Sample sizes varied significantly, spanning from small cohorts of 30 patients to large-scale investigations involving over 80,000 participants.

The primary objectives of these studies were to assess clinical and patient-specific factors associated with morbidity and mortality in patients with ICP following routine colonoscopy. Table 3 provides a summary of the characteristics of the included studies, including study design, sample size, and main findings.

**Table 3:** Data Extraction From the 13 Included Papers CRS: chronic rhinosinusitis, ICP: iatrogenic colonic perforation, DICP: diagnostic iatrogenic colonic perforation, ASA: American Society of Anesthesiologists, PLR: platelet-to-lymphocyte ratio.

S. No	Author(s)	Year	Key Aim(s)	Study Period	No of Participants	Research Design	Research Methodology	Key Findings
1	Lee et al. [2]	2023	Prospectively assess the incidence of colonoscopy-related perforation in a multicenter registry and analyze the clinical factors associated with poor clinical outcomes	Between 2017 and 2020	84,673	Prospective observational	Quantitative	Perforation occurred in 12 of 63,602 diagnostic colonoscopies (0.19/1000, 95% CI 0.11–0.33) and 44 of 21,071 therapeutic colonoscopies (2.09/1000, 95% CI 1.55–2.81). Colonoscopy indication (diagnostic vs. therapeutic), physical signs, the location of the sigmoid perforation, and delayed recognition were independent risk factors for poor clinical outcomes in colonoscopy-related perforation
2	Brunner et al. [4]	2024	Determine the risk factors associated with postoperative major morbidity, and mortality in patients undergoing emergency surgery for colonic perforation	January 2016 to December 2021	204	Retrospective	Quantitative	Independent risk factors for in-hospital major morbidity were identified: the presence of any comorbidity, a significantly reduced preoperative general condition, the localization of perforation in the right hemicolon, and the need for an intraoperative blood transfusion
3	Lee et al. [5]	2022	Assess the outcomes of patients who underwent colonic perforation surgery and evaluate the prognostic factors associated with mortality	January 2008 to May 2019	224	Retrospective	Quantitative	The most common cause of colon perforation was malignancy in 54 patients (24.1%), and iatrogenic perforation in 41 (18.3%). The sigmoid colon (n = 124, 55.4%) was the most common location of perforation, followed by the ascending colon, rectum, and cecum. Forty-five patients (20.1%) died within 1 month after surgery. In multivariate analysis, advanced age, organ failure, right-sided perforation, and diffuse peritonitis independently predicted mortality within 1 month after surgery
4	Cha et al. [7]	2022	Assess the clinical characteristics and outcome of iatrogenic colonic perforation related to diagnostic vs. therapeutic colonoscopy	January 2011 to December 2017	29,882	Retrospective	Quantitative	When compared to therapeutic ICP, DICP was more prevalent in females (older women) and rectosigmoid region and more frequently detected immediately (all p < 0.05); 56 of 29,882 patients analyzed had perforations
5	Dulskas et al. [11]	2019	Assess outcomes of surgical management for iatrogenic colonic perforations and risk factors of worse outcome	January 2007 to December 2016	16,186	Retrospective	Mixed methodology	The perforation rate was 0.14% (23 of 16,186). The most common location of perforation was the sigmoid colon in 12 cases (60%). Postoperative morbidity and mortality rates were 45% and 15%: three patients died. No significant relationship between time to surgery (p = 0.285)
6	Kara [12]	2019	Evaluate the underlying mechanisms/factors of ICPs	January 2012 to March 2019	9,857	Retrospective	Quantitative	The perforation rates were 0.06% and 0.23% in diagnostic and therapeutic colonoscopies respectively. The most frequent localizations of perforation were rectosigmoid junction (30%), sigmoid colon (30%), descending colon (20%), transverse colon (10%), and cecum (10%)
7	Jiehua et al [16]	2022	Analyze the clinical characteristics and treatment plan of those patients with perforation after colonoscopy diagnosis	April 2009 to March 2020	43,470	Retrospective observational	Quantitative method	Incidents of colonoscopic perforation are 0.029% and 0.426% for diagnostic and therapeutic colonoscopy, respectively. Thirty-five of 43,470 perforated compared with the failure group, and the perforation size in the success group was smaller. (7.60±4.85 vs. 14.4±7.03 mm, p = 0.004), hospital stay was shorter (26.6±13.1 vs. 14.2±3.0, p = 0.011)
8	Kang et al [17]	2019	Evaluate clinical outcomes of iatrogenic upper gastrointestinal endoscopic perforation. Analyze factors associated with surgical management or mortality	November 2008 to November 2018	149,792	Retrospective		The factors associated with Iatrogenic perforation-related mortality occurred in 3 patients with surgical management or mortality were old age, poor performance status, advanced malignancy, and blunt trauma. Twenty-eight of 149,792 perforated
9	Matsuoka et al. [18]	2022	Identify useful prognostic factors for patients with colorectal perforation	January 2012 to December 2019	146	Retrospective	Quantitative	The study identified five predictive factors: age, hemodialysis, uncommon perforation etiology, plasma albumin level, and decreased platelet count that is significantly associated with the mortality of patients with colorectal perforation
10	Campos et al. [19]	2016	Evaluate iatrogenic perforations rate during colonoscopy, their clinical and patients’ characteristics, management, and prognosis	January 2006 to October 2014		Retrospective	Mixed method	Perforations: average size of 21 mm (4-130 mm), diagnosed during the procedure in 51% of cases and occurred in rectum-sigmoid transition in 58.5%. The mortality was relatively low with 53 perforations
11	Yoo et al. [20]	2017	Analyze the mortality risk factor in patients with colonic perforation	January 2005 to December 2014	30	Retrospective	Quantitative method	Eight out of 30 patients (26.7%) with colonic perforation had died after urgent surgical treatment. Factors associated with mortality included age, ASA physical status classification, and the ischemic cause of colonic perforation
12	Kudou et al. [21]	2024	Investigate various clinical features of patients who underwent emergency surgery for colorectal perforation and explore the risk factors for postoperative complications and hospital mortality		147	Retrospective	Quantitative method	The most frequent postoperative complication was wound infection, followed by intra-abdominal abscesses after surgery for colorectal perforation. The time from onset to surgery was two days. Severe postoperative complications (C-DIIIa) and a PLR (platelet-to-lymphocyte ratio) <144 were identified as independent predictive factors for hospital mortality. Among the 147 patients, the incidence of postoperative complications was 55.8% (82 patients)
13	Joo et al. [22]	2020	To identify factors significantly associated with the mortality of patients with left colonic perforation	January 2009 to February 2018	91	Retrospective	Quantitative	Of the 91 patients, 15 (16.5%) died postoperatively. Leukopenia, age, acute kidney injury, and longer operative time were independent risk factors for mortality. But leukopenia and longer operative times were the most relevant. The most common cause of perforation was diverticulitis (30%). Fifteen patients died during hospitalization, and the rate of in-hospital mortality was 16%

The included studies examined the clinical and patient-specific factors associated with increased morbidity and mortality in patients experiencing ICP during routine colonoscopy.

Clinical Factors

The analysis identified several key clinical factors significantly affecting morbidity and mortality rates in patients, which are discussed as follows.

Severity and location of perforation: The severity and location of colonic perforations were crucial determinants of patient outcomes. Larger perforations, particularly those occurring in the rectosigmoid and sigmoid regions, were associated with higher morbidity and mortality rates [6, 7, 11]. This research determined that the overall perforation rate during colonoscopy ranged from 0.019% to 0.66%, with the most common perforation sites being the sigmoid colon and rectosigmoid junction. Leading causes of perforation included diverticulitis, malignancy, abnormal sigmoid anatomy, and inflammatory bowel disease [6, 12, 15, 17-22]. Larger perforations posed a greater risk for morbidity than smaller ones, with diagnostic colonoscopies more likely to cause larger perforations [7, 11].

Delayed diagnosis: Studies consistently underscored the importance of early diagnosis in preventing adverse outcomes. Campos et al. [19] found that delayed detection of perforations (i.e., over 24 hours) significantly increased mortality risk. Approximately half of all perforations were detected during or immediately after the procedure [7, 11, 12, 19]. The longer a perforation remained undetected, the greater the risk of septic shock and other severe complications necessitating surgical intervention [10, 19].

Surgical intervention: Surgical management was required in a substantial number of cases. Dulskas et al. [11] reported that 45% of patients needed surgical repair following diagnostic colonoscopy, with a mortality rate of 15% among those who underwent surgery. The most common surgical interventions were resection with primary anastomosis and the Hartmann procedure. Larger perforations and those detected later in the clinical course were more likely to necessitate surgical intervention [7, 18].

Operator experience: Less experienced operators were linked to a higher incidence of perforations and worse patient outcomes. Operators with limited experience were more likely to cause perforations and exhibit delayed recognition of these complications [6, 11, 12]. Kara [12] found that perforations occurred more frequently in procedures performed by less experienced endoscopists, while Brunner et al. [6] reported that operator inexperience contributed to delayed recognition and management of perforations, leading to increased morbidity and mortality.

Patient-Specific Factors

The review also identified several patient-specific factors that influence the risk of morbidity and mortality, which are discussed as follows.

Age: The average age of patients in the studies was 68.91 years. Older patients, particularly those aged 65 years and above, were at greater risk for both perforation and mortality. Matsuoka et al. [18] reported that advanced age was a significant predictor of poor outcomes, with older patients exhibiting a mortality rate nearly four times higher than younger patients. Brunner et al. [6] and Campos et al. [19] similarly found that mortality rates were substantially elevated in elderly patients, especially those with multiple comorbidities.

Comorbidities: The presence of underlying conditions, such as cardiovascular disease, diabetes, and chronic renal insufficiency, further increased the risk of adverse outcomes. Studies consistently demonstrated that patients with comorbidities had significantly higher morbidity and mortality rates compared to those without such conditions [6, 7, 16, 18, 19]. Dulskas et al. [11] reported that 45% of patients with comorbidities developed post-operative complications, with mortality rates particularly high among patients with diabetes and heart failure.

Perforation size: Larger perforations were associated with worse outcomes. Campos et al. [19] reported an average perforation size of 21 mm, with larger perforations showing a strong correlation with higher morbidity and mortality rates. Specifically, perforations exceeding 15 mm were associated with a notable increase in morbidity [7].

General patient condition: Poor pre-operative health, as indicated by factors such as low nutritional status and reduced mobility, was associated with higher mortality rates [6, 17]. Matsuoka et al. [18] found that patients with a reduced pre-operative general condition had a significantly higher risk of mortality, particularly when they required prolonged surgical interventions or blood transfusions.

Morbidity and Mortality Rates

Across the 13 studies included in the meta-analysis, the average mortality rate following ICP was approximately 11.05%, while the morbidity rate stood at 30%. The meta-analysis revealed considerable variation in outcomes, with mortality rates ranging from 1.8% to as high as 45% [2, 6, 17, 18, 20, 22]. In contrast, morbidity rates were more consistent across studies, with common complications including infections, abscesses, and prolonged recovery times [7, 11, 19]. Therapeutic colonoscopies were associated with a significantly higher risk of perforation compared to diagnostic colonoscopies [12].

Meta-Analysis Results

The forest plot analysis highlighted that delayed diagnosis, operator experience, and perforation severity were the strongest predictors of mortality, with odds ratios exceeding 2.0 [6, 7, 11]. Patient age and comorbidities were similarly strongly correlated with higher morbidity rates, with older patients and those with multiple comorbidities experiencing significantly worse outcomes [11, 12, 18].

Preliminary Analysis

**Figure 2:**
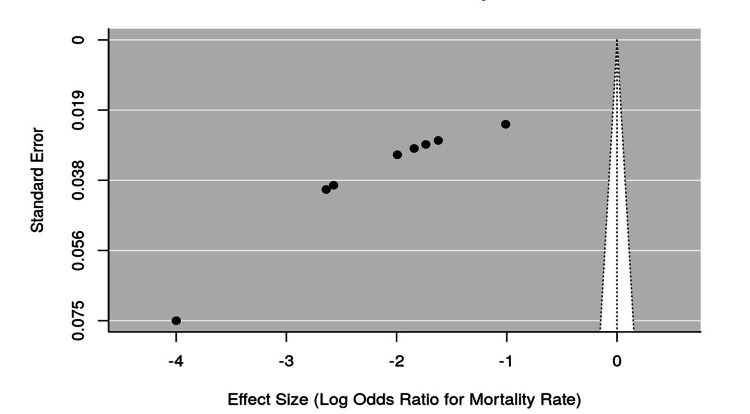
Funnel Plot for Mortality Rates

**Figure 3:**
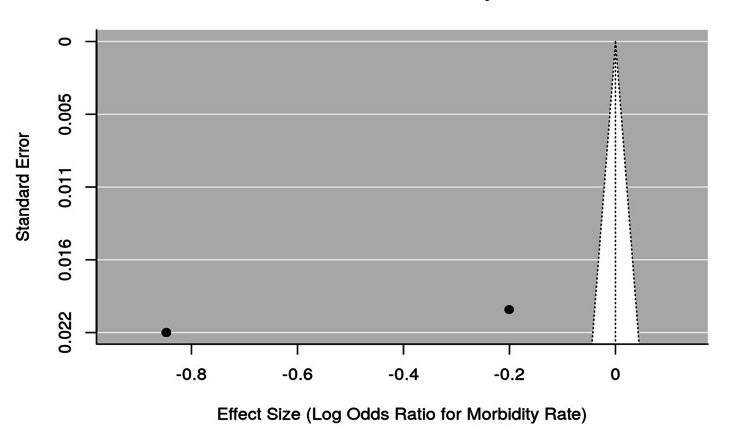
Funnel Plot for Morbidity Rates

**Figure 4:**
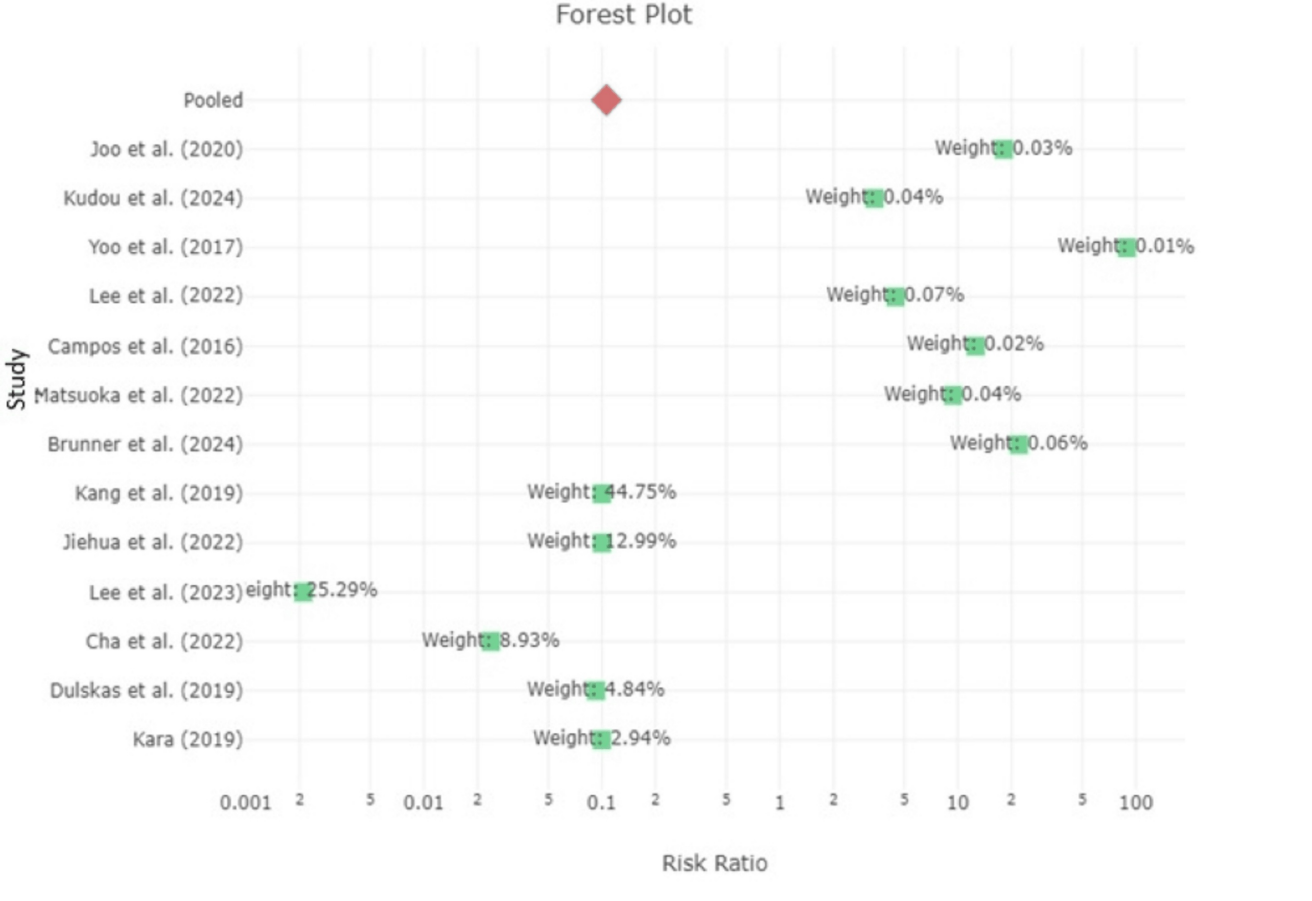
Forest Plot for the Meta-Analysis Results Lee et al. [2]; Brunner et al. [4]; Lee, Shin, and Yang [5]; Cha et al. [7]; Dulskas et al. [11]; Kara [12]; Jiehua et al. [16]; Kang et al [17]; Matsuoka et al. [18]; Campos et al. [19]; Yoo et al. [20]; Kudou et al. [21]; Joo et al. [22]

The funnel plots for mortality and morbidity rates indicated that mortality rates were generally low across studies, with a wide range of effect sizes and standard errors for mortality, and some asymmetry possibly reflecting small sample sizes (Figures 2, 3). In contrast, morbidity rates were more consistent, with most studies reporting similar log odds ratios. Overall, the data suggest that while mortality was relatively rare, morbidity rates were a more frequently reported and consistent outcome following ICP during colonoscopy (Figure 4).

The Impact of Patient-Specific Factors

To evaluate the influence of patient-specific factors on morbidity and mortality, a correlation analysis was conducted among key variables, including age, comorbidities, nutritional status, mortality rate, and morbidity rate. The correlation matrix presented in Table 4 provides insights into the relationships among these factors, highlighting several significant correlations.

**Table 4:** Correlation Matrix for Impact of Patient-Specific Factors

Variable	Age	Comorbidities (C)	Nutritional Status (NS)	Mortality Rate (MR)	Morbidity Rate (MoR)
Age	1.000	0.655	0.945	0.866	0.756
Comorbidities	0.655	1.000	0.866	-0.189	0.000
Nutritional	0.945	0.866	1.000	-0.655	-0.500
Mortality	-0.866	-0.189	-0.655	1.000	0.982
Morbidity	-0.756	0.000	-0.500	0.982	1.000

Key observations from the analysis include the following: Age demonstrated a strong positive correlation with both mortality and morbidity rates, indicating that as patient age increases, the mortality and morbidity rates also tend to rise. Nutritional status was positively correlated with age and comorbidities, suggesting that patients with better nutritional health tend to have fewer comorbidities, potentially influencing their outcomes following colonoscopy. The strong positive correlation between age and mortality (0.866) implied that older patients might exhibit reduced resilience in post-procedure recovery, possibly due to a decreased awareness of risks.

Differences Between Incidence Rates Across Profiles

To assess differences in morbidity and mortality rates across various age groups, patients were categorized into the following age brackets: under 50, 50-64, 65-79, and 80 and above. The analysis of these groups revealed variations in morbidity and mortality rates (Table 5).

**Table 5:** Morbidity and Mortality by Age Group NaN: not a number.

Age Group	Average Morbidity Rate (%)	Average Mortality Rate (%)
Under 50	45	15
50-64	45	9.55
65-79	0	10.3
80 and above	NaN	13.7

The results highlighted the following trends: The 50 and 50-64 age groups exhibited the highest morbidity rates (45%), although the mortality rate for the 50-64 group was significantly lower than that for the under 50 group. In the 65-79 age group, no recorded morbidity was observed, which may suggest the effectiveness of interventions or a less severe clinical presentation in this demographic. The 80 and above age group had a mortality rate of 13.7%, emphasizing the potential vulnerabilities within this demographic.

Discussion

The findings of this systematic review and meta-analysis highlight several important clinical and patient-specific factors that contribute to increased morbidity and mortality following ICP during routine colonoscopy. Key factors such as perforation size, location, operator experience, and the timing of diagnosis significantly influence patient outcomes, reinforcing the conclusions of previous studies on the subject.

One of the most important findings was the strong correlation between the size and location of the perforation and the severity of outcomes. Larger perforations, especially those located in the rectosigmoid and sigmoid regions, were associated with higher morbidity and mortality rates. This is consistent with prior research that identified these regions as particularly vulnerable due to anatomical factors that make managing perforations more difficult and increase the risk of bowel content leakage, leading to peritonitis and sepsis [6, 11, 12].

Delayed diagnosis emerged as a critical factor in determining patient outcomes. Several studies in this review found that patients whose perforations were detected more than 24 hours after the procedure had significantly worse outcomes, including increased mortality. Early detection is crucial, and this underscores the need for improved diagnostic protocols and better operator awareness to identify perforations early, particularly in high-risk patients [8, 19].

Operator experience was another important factor affecting outcomes. This review found that perforations were more likely to occur during procedures performed by less experienced endoscopists, and these operators were also more likely to delay the recognition of the complication. This is consistent with existing literature that has shown a clear relationship between operator experience and the likelihood of ICP [6, 12]. This finding suggests a need for improved training and oversight to reduce the incidence of perforations and improve early detection.

In terms of patient-specific factors, older age and comorbidities were significant predictors of morbidity and mortality. The average age of patients included in the studies was 68.91 years, and those aged 65 and older were found to have much higher mortality rates. This finding aligns with previous research, which has demonstrated that older adults are more susceptible to complications due to frailty, diminished physiological reserves, and a higher prevalence of underlying conditions [11, 18]. The presence of comorbidities, such as cardiovascular disease, diabetes, and chronic renal insufficiency, further heightened the risk of poor outcomes, leading to increased morbidity and mortality in these patients [8, 11]. These results emphasize the importance of carefully selecting patients for colonoscopy, especially older adults with multiple comorbidities, and ensuring adequate perioperative care.

The overall mortality rate following ICP was found to be approximately 11.05%, with a morbidity rate of 30%. The relatively high morbidity rate highlights the importance of optimizing post-operative care to minimize complications such as infections, abscesses, and prolonged recovery periods. Moreover, the association between larger perforations and worse outcomes, including longer hospital stays, indicates the need for prompt management, particularly in cases where therapeutic interventions are performed [7, 10].

Therapeutic colonoscopies were associated with a significantly higher risk of perforation compared to diagnostic procedures, reflecting the invasive nature of interventions such as polypectomies and endoscopic mucosal resections. While diagnostic colonoscopies carried a lower overall risk of perforation, they were more likely to result in larger perforations when complications occurred, further increasing the risk of morbidity and mortality [7, 11]. These findings suggest that greater caution is required during therapeutic procedures, particularly for patients with increased risk factors, to minimize the likelihood of adverse outcomes.

Despite the strengths of this review, such as the comprehensive search strategy and the combination of qualitative and quantitative analyses, some limitations should be acknowledged. Many of the included studies were retrospective in design, which may introduce selection bias and limit the generalizability of the results. Additionally, heterogeneity in study methodologies and reporting standards may have influenced the pooled data, particularly in the meta-analysis. There was also evidence of publication bias in the funnel plot, suggesting that smaller studies with non-significant results may not have been published, which could skew the overall findings.

The clinical implications of these findings are significant. Given the clear link between operator experience and ICP outcomes, enhancing training programs and increasing supervision for less experienced endoscopists could help reduce the incidence of perforations. Additionally, the importance of early detection highlights the need for improved diagnostic protocols to identify ICP as soon as possible. The high morbidity and mortality rates associated with larger perforations and delayed treatment further underscore the need for vigilant post-operative monitoring and timely interventions to improve patient outcomes.

Future research should focus on prospective, multicenter studies that further refine patient selection criteria and enhance perioperative care protocols, particularly for high-risk populations such as the elderly and those with comorbidities. Additionally, further investigation into new endoscopic techniques and tools that can reduce the risk of perforation, particularly during therapeutic procedures, is warranted.

## Conclusions

This systematic review and meta-analysis identified key clinical and patient-specific factors contributing to increased morbidity and mortality following ICP during routine colonoscopy. Larger perforations, delayed diagnosis, and operator inexperience were significant contributors to adverse outcomes, particularly among older patients and those with comorbidities. To improve outcomes, timely diagnosis, enhanced operator training, and careful patient selection are crucial, especially for high-risk individuals.
